# The Role of ICS/LABA Fixed-Dose Combinations in the Treatment of Asthma and COPD: Bioequivalence of a Generic Fluticasone Propionate-Salmeterol Device

**DOI:** 10.1155/2021/8881895

**Published:** 2021-03-17

**Authors:** Donald P. Tashkin, Jill A. Ohar, Arkady Koltun, Richard Allan, Jonathan K. Ward

**Affiliations:** ^1^Department of Medicine, David Geffen School of Medicine at UCLA, Los Angeles, CA, USA; ^2^Department of Internal Medicine, Section of Pulmonary, Critical Care, Allergy and Immunologic Medicine at Wake Forest University School of Medicine, Winston-Salem, NC, USA; ^3^Viatris/Mylan Inc., Canonsburg, PA, USA; ^4^Viatris/Mylan Pharma UK Ltd., UK

## Abstract

Both asthma and chronic obstructive pulmonary disease (COPD) are inflammatory chronic respiratory conditions with high rates of morbidity and mortality worldwide. The objectives of this review are to briefly describe the pathophysiology and epidemiology of asthma and COPD, discuss guideline recommendations for uncontrolled disease, and review a new generic option for the treatment of asthma and COPD. Although mild forms of these diseases may be controlled with as-needed pharmacotherapy, uncontrolled or persistent asthma and moderate or severe COPD uncontrolled by bronchodilators with elevated eosinophilia or frequent exacerbations may require intervention with combination therapy with inhaled corticosteroids (ICS) and long-acting beta agonists (LABAs), according to international guidelines. Fixed-dose combinations of ICS/LABA are commonly prescribed for both conditions, with fluticasone propionate (FP) and salmeterol forming a cornerstone of many treatment plans. An oral inhalation powder containing the combination of FP and salmeterol has been available as Advair Diskus® in the United States for almost 20 years, and the first and only substitutable generic version of this product has recently been approved for use: Wixela™ Inhub™. Bioequivalence of Wixela Inhub and Advair Diskus has been established. Furthermore, the Inhub inhaler was shown to be robust and easy to use, suggesting that Wixela Inhub may provide an alternative option to Advair Diskus for patients with asthma or COPD requiring intervention with an ICS/LABA.

## 1. Introduction

One of the leading causes of morbidity and mortality worldwide is chronic respiratory disease, of which chronic obstructive pulmonary disease (COPD) and asthma are 2 of the most common [1]. Although asthma and COPD may have overlapping characteristics, they have distinct etiologies, presentations, and pathophysiologies (Table 1) [2, 3].

According to the 2020 Global Initiative for Chronic Obstructive Lung Disease report, COPD is a chronic, preventable condition characterized by “persistent respiratory symptoms and airflow limitation that is due to airway and/or alveolar abnormalities usually caused by significant exposure to noxious particles or gas” [2]. Common respiratory symptoms of COPD include dyspnea, cough, sputum production, wheeze, and chest tightness. By contrast, asthma is a heterogeneous condition characterized by a “history of respiratory symptoms such as wheeze, shortness of breath, chest tightness and cough that vary over time and in intensity, together with variable expiratory airflow limitation,” according to the Global Initiative for Asthma [3].

The prevalence of asthma and COPD and their clinical sequelae have increased over the past 2 decades. Worldwide, more than 500 million people have COPD, asthma, or both. Furthermore, worldwide in 2015, 3.2 million people died from COPD, and 0.40 million died from asthma [4]. In addition to premature mortality, substantial morbidity has been attributed to these conditions, with up to 26% of patients with COPD visiting the emergency department or being hospitalized in a year [5, 6]. Similarly, asthma is the third leading cause of hospitalizations in children in the United States and is present in 10.1% of all individuals visiting the emergency department [7, 8].

Although asthma and COPD are complex diseases with a multitude of treatment options, the majority of patients with these conditions are diagnosed and treated by primary care providers (PCPs). In a telephone-based survey of 2500 patients with asthma, 48% had never visited an asthma specialist, and three-quarters had not used a specialist for usual asthma care [9]. The objective of the present review is to describe unmet clinical needs in asthma and COPD, discuss asthma and COPD guidelines, and review a new generic combination treatment option for these complex conditions, with a focus on clinical aspects of importance to PCPs.

## 2. Asthma and COPD Pathophysiology

Both asthma and COPD are inflammatory respiratory disorders characterized by partially related clinical and pathophysiologic changes. The similar symptoms of asthma and COPD can make it challenging to distinguish between the diseases. Doing so is a critical component of diagnosis, because of the distinct disease progression and management strategies for these 2 conditions [10].

Asthma symptoms are mediated by airway narrowing caused by an interplay of airway hyperresponsiveness, inflammation, structural changes (remodeling), and environmental exposures [11]. Inflammatory cells vary by individual and can include mast cells, eosinophils, T-cells, macrophages, and neutrophils [7]. Structural changes include subepithelial fibrosis, increased airway smooth muscle, increased vascularity in airway walls, and mucus hypersecretion [7]. These pathologic changes lead to airway narrowing, which is caused by airway smooth muscle contraction, airway edema, airway wall thickening, and mucus hypersecretion. Airway narrowing itself is also a signal for continued structural remodeling, resulting in a cyclic process of inflammation, hyperresponsiveness, airway narrowing, and remodeling [7].

In contrast to asthma, COPD symptoms are caused by narrowing of peripheral airways and regional areas of air trapping, leading to ventilation-perfusion mismatch and limiting expiratory flow. Airway narrowing is caused by an interplay of chronic inflammation, parenchymal tissue destruction, and small airway fibrosis [2]. An emphysematous component of COPD also limits expiratory flow through destruction of the alveolar walls, which also leads to impairment in gas transfer, further contributing to the symptoms of COPD [12]. Cigarette smoke inhalation or inhalation of other noxious stimuli, along with genetic predisposition, is believed to cause the lung inflammation characteristic of COPD [12]. Inflammation is primarily driven by elevated levels of alveolar macrophages, neutrophils, T-cells, and innate lymphoid cells [13], as well as eosinophils in a subset of patients [13, 14].

## 3. Unmet Clinical Needs in Asthma and COPD

A shared characteristic of asthma and COPD is a population-level lack of symptom control, nonadherence to prescribed therapy, and the frequent occurrence of exacerbations [9, 15]. In patients with asthma, control is measured by the frequency of symptoms and need for rescue medication, as well as the future risk for adverse outcomes (e.g., exacerbations) [3, 15]. According to a report by the National Asthma Education and Prevention Program, up to two-thirds of patients with asthma have uncontrolled disease [9].

Furthermore, guideline-directed asthma management is lacking in the United States. Nearly 90% of people with asthma have disease that is considered mild or worse [16]. If all patients with asthma were receiving guideline-directed treatment, 90% would be using daily controller medication; however, only 22% of surveyed patients with asthma were using daily long-term controller medication [15].

Similar results have been reported for patients with COPD. In the United States, 26% of patients with COPD visited the emergency department in the preceding 12 months, and 17% were hospitalized [6]. Furthermore, about two-thirds of all patients with COPD are not prescribed maintenance therapy, and approximately 10% receive no medication at all [17].

Both patients and physicians have been shown to overestimate disease control and underestimate exacerbations. For example, patients considered asthma well managed if rescue medication was used 3 times per week, urgent care visits occurred twice per year, or emergency department visits occurred once per year [9]. Physicians have also been shown to routinely underestimate the impact of COPD on their patients' lives [18]. These data underscore the need for improved adherence to standard-of-care maintenance therapy for patients with chronic respiratory illnesses.

## 4. Standards of Care in Asthma and COPD

Asthma treatment should be escalated in a stepwise manner as needed to address the domains of control and severity [3, 19]. Some patients with asthma may be controlled with as-needed or daily low-dose inhaled corticosteroid (ICS), but patients with persistent asthma symptoms occurring most days or with nighttime waking once or more per week may require step-up therapy to daily combination treatment with low- or medium-dose ICS/long-acting beta agonist (LABA) combinations. Escalating controller medication to daily combination therapy may be particularly important for patients with risk factors for poor asthma outcomes (Table 2) [3, 19].

ICS and LABAs can be administered individually or by fixed-dose combination inhalers. Currently approved combination ICS/LABA inhalers for asthma include fluticasone propionate (FP)-salmeterol, FP-formoterol, beclomethasone-formoterol, budesonide-formoterol, and mometasone-formoterol, all of which are administered twice daily. Furthermore, most recently introduced is fluticasone furoate-vilanterol, which is administered once daily [3].

Pharmacologic management of COPD should be individualized based on symptom severity, exacerbation risk, adverse events, comorbidities, drug availability, and cost, as well as patient response, preference, and ability to use inhalers properly. An ICS combined with a LABA has been shown to improve lung function and reduce exacerbations relative to either drug alone [20]. This combination therapy is recommended for patients with moderate-to-very-severe COPD with high eosinophil levels, or a history of 2 or more moderate exacerbations or at least 1 severe (hospitalized) exacerbation [2]. A firm cutoff for elevated blood eosinophils in COPD has not been established, but the consensus is that measurements of 150 cells/*μ*L to ≥300 cells/*μ*L are considered elevated [21]. Also of note, if more than 1 eosinophil measurement is available, all recorded measurements should be taken into consideration, because systemic glucocorticoid therapy can affect blood eosinophil count [22].

## 5. FP-Salmeterol Combination in Asthma and COPD

FP-salmeterol dry powder for oral inhalation is a fixed-dose combination ICS/LABA that is widely prescribed for both asthma uncontrolled with low-dose ICS alone and for symptomatic COPD with frequent exacerbations and/or elevated blood eosinophil counts [23, 24]. The addition of salmeterol to FP in a combination inhaler has been shown to significantly reduce the risk for severe asthma exacerbations by 21% compared with FP alone (*P* < 0.001) [25]. According to the results of a Cochrane systematic review of 14 studies with 11,794 participants with severe COPD, fixed-dose ICS/LABA combination inhalers were associated with a 24% lower risk for exacerbation compared with a LABA alone, but studies showed statistical heterogeneity [20].

In the European Union, FP-salmeterol dry powder inhaler (DPI) is marketed as Seretide Accuhaler/Diskus®, while in the United States, FP-salmeterol inhalation powder is marketed as Advair Diskus® (GlaxoSmithKline, Research Triangle Park, NC) [24, 25]. The patent for Advair Diskus expired in 2016. In January 2019, a generic formulation of FP-salmeterol DPI, Wixela™ Inhub™ (Mylan, Morgantown, WV), was approved for use as a substitutable generic equivalent of Advair Diskus by the US Food and Drug Administration (FDA) for the maintenance treatment of asthma and COPD [26]. Generic medicines provide the same clinical benefits as brand name products in terms of dose, safety, efficacy, stability, and quality. Furthermore, compared with brand name medications, generic medications have been shown to provide cost-savings to healthcare systems and to facilitate patient access, with little to no impact on adherence to this important maintenance treatment for asthma and COPD [27, 28].

Another advantage of generic medications is the potential for innovation in drug delivery technology [29]. Like the Diskus, the Inhub DPI holds 60 doses of 100 *μ*g, 250 *μ*g, or 500 *μ*g FP and 50 *μ*g salmeterol per dose (i.e., 100/50 *μ*g, 250/50 *μ*g, and 500/50 *μ*g) [25, 30]. The key steps required to use the Inhub are as follows: open the mouthpiece, push down the lever, exhale as long as possible (not into the inhaler), inhale quickly and forcefully, hold breath, and close the mouthpiece [30]. These steps are comparable with those of the Diskus, with the main exception being the orientation of the inhaler during operation [25]. The Inhub inhaler is held vertically with the lever pushed down, whereas the Diskus is held horizontally with its lever slid around the Diskus [25, 30]. The Inhub can be operated with either hand, whereas the Diskus was developed to be held in the left hand [25, 30]. The Inhub is considered to have comparable resistance to the Diskus; most adult and pediatric patients with asthma and those with severe COPD are able to achieve a peak inspiratory flow rate of >30 L/min through the inhaler [31].

## 6. Generic FP-Salmeterol Comparison with Branded FP-Salmeterol Combination

Generic medications must undergo rigorous testing to show they can effectively substitute for a brand name medication [27]. Approval of generic drugs by the FDA requires demonstration of both pharmaceutical equivalence and bioequivalence. Pharmaceutical equivalence is defined by the FDA as “drug products in identical dosage forms and route(s) of administration that contain identical amounts of the identical active drug ingredient,” while bioequivalence is defined by the FDA as “absence of a significant difference in the rate and extent to which the active ingredient or active moiety in pharmaceutical equivalents or pharmaceutical alternatives becomes available at the site of drug action when administered at the same molar dose under similar conditions in an appropriately designed study” [32]. For inhaled drugs, the demonstration of bioequivalence is challenging due to the localized site of action. Therefore, the FDA utilizes an “aggregate weight-of-evidence” approach, which includes the following 3 types of studies: in vitro studies, pharmacokinetic studies, and pharmacodynamic or clinical endpoint studies. Together, these studies meet the FDA's aggregate weight-of-evidence approach and can demonstrate bioequivalence [33–35].

Wixela Inhub was compared with Advair Diskus, and the 2 products were shown to be bioequivalent using clinical endpoints in a large double-blind, placebo-controlled trial of 1127 adults with mild to moderate, persistent asthma [36]. In this study, participants were randomly assigned to receive Wixela Inhub (100/50 *μ*g), Advair Diskus (100/50 *μ*g), or placebo twice daily for 28 days. Lung function was assessed using spirometry, and forced expiratory volume in 1 second (FEV_1_) was recorded and compared across treatment groups, with coprimary endpoints including measures on days 1 and 29. On day 1, FEV_1_ area under the response-time curve (AUC) over 12 hours of the change from baseline was significantly improved compared with placebo in both the Wixela Inhub group (3.134 L hr; *P* < 0.0001) and the Advair Diskus group (2.677 L hr; *P* < 0.0001). Furthermore, on day 29, both treatment groups had increased trough FEV_1_ compared with placebo (Wixela Inhub, 235 mL; Advair Diskus, 215 mL; each *P* < 0.0001). These endpoints were tested in a bioequivalence comparison, with Advair Diskus as the reference product and Wixela Inhub as the test product. For both endpoints, the geometric mean ratio (test : reference) and associated upper and lower 90% confidence intervals (CIs) were within the permissible range of 0.80 to 1.25. These results provided evidence that Wixela Inhub and Advair Diskus had local pulmonary therapeutic equivalence of FP-salmeterol components after inhalation [36].

The systemic exposure of Wixela Inhub and Advair Diskus was compared in a separate program of 3 pharmacokinetic bioequivalence studies. In each open-label, crossover study, 66 healthy participants performed 3 inhalations of 1 of the 3 dose strengths of FP-salmeterol (100/50 *μ*g, 250/50 *μ*g, or 500/50 *μ*g) delivered using the Advair Diskus or Wixela Inhub, and plasma levels of FP and salmeterol were measured over 48 hours postdose [37]. Pharmacokinetic parameters (maximum plasma concentration and AUC) were compared between the products. Estimated geometric mean ratios and associated 90% CIs for the AUC plasma concentrations from time 0 to the last measurable concentration ranged from 0.97 to 1.07 for FP components and from 1.00 to 1.08 for salmeterol components across dose ranges. Similarly, estimated geometric mean ratios and 90% CIs for the maximum observed plasma concentration ranged from 0.90 to 1.01 for FP and from 0.86 to 1.00 for salmeterol components across dose ranges. These values were within the prespecified bioequivalence acceptance criterion of 0.80 to 1.25. At all doses, FP-salmeterol was well tolerated via both Wixela Inhub and Advair Diskus [37].

With the modest difference in operating characteristics of the Inhub inhaler relative to Diskus, evaluation of the robustness and ease of use was an important component of the premarket investigations. Robustness was assessed by analyzing the in vitro integrity and performance of FP and salmeterol remaining in the inhaler after outpatient use. In an open-label study, patients with asthma (≥12 years of age) or with COPD (≥40 years of age) used the Inhub for 3 weeks. After outpatient use, comprehensive physicochemical and microbiological integrity testing of the product remaining in unused pockets of the inhaler included in vitro microbiology, water content, assay, emitted dose content uniformity, aerodynamic particle size distribution, and degradation tests. The results of the in vitro testing demonstrated that the pharmaceutical integrity and performance of both FP and salmeterol were preserved [38].

Correct use of an inhaler is critical to maintaining disease control, underscoring the importance of ease-of-use testing. In another open-label study, 110 patients with asthma, COPD, or both were recruited as part of 2 groups: those who had used Diskus before and those who had never used a DPI (DPI-naïve patients). Participants included adolescents, the elderly, and people with manual dexterity issues. At the start of the study, participants were provided with the Inhub and packaging representative of commercial packaging. Use of the Inhub was monitored, and the vast majority of subjects used the Inhub correctly, following the steps outlined in the instructions for use, including holding the Inhub in the correct orientation [38].

Despite the extensive bioequivalence evaluation of Wixela Inhub, several questions may remain to be fully answered. The clinical endpoint bioequivalence study of Wixela Inhub did not enroll patients with COPD and used only the 100/50 *μ*g dose [36]. It should be noted that, according to product-specific FDA guidance [34], clinical endpoint bioequivalence does not have to be demonstrated with all 3 strengths of the product or in every proposed indication (i.e., asthma and COPD) and can instead be demonstrated using the lowest approved strength of the product (i.e., 100/50 *μ*g) and in the most clinically responsive patient population (i.e., asthma). This recommendation reflects that any reduction in efficacy of the generic product would be most evident in the population that is the most clinically sensitive to the product class, given that the dose-response of the ICS component is very shallow, with the majority of the clinical benefit being achieved at the lowest approved doses [30]. Furthermore, the demonstration of equivalent performance in vitro and, in particular, pharmacokinetic bioequivalence of all 3 product strengths provides further assurance of equivalent systemic and local lung delivery of the 2 products, especially given that FP has low oral bioavailability and that the systemic pharmacokinetics reflect absorption of the lung deposited dose [39]. Although there is no evidence that the bioequivalence of Wixela Inhub will vary according to patient disease state or dose, more extensive research would be needed to verify this. Additional head-to-head studies could also be beneficial to evaluate patient preference and satisfaction.

## 7. Conclusions

Persistent asthma uncontrolled with low-dose ICS alone and moderate-to-very-severe COPD with elevated eosinophilia or frequent exacerbations may need intervention with ICS/LABA. Fixed-dose combination with an FP-salmeterol DPI is a common option that was only available under the brand Advair Diskus until recently. Wixela Inhub is a generic formulation of FP-salmeterol that became available in 2019. Premarketing studies of Wixela Inhub have comprehensively demonstrated bioequivalence to Advair Diskus, and human factor research has indicated that the Inhub is robust and easy to use. Wixela Inhub is the first and only product available on the US market as a substitutable generic equivalent of Advair Diskus, thus providing a cost-effective alternative.

## Figures and Tables

**Table 1:** Differences between asthma and COPD presentation, etiology, and pathophysiology [2, 3].

	Asthma	COPD
Characteristic symptom	Variable (i.e., wheezing, shortness of breath, chest tightness, or cough may be the prominent symptom, depending on the patient)	Chronic and progressive dyspnea, particularly during exertion
Other symptoms	Wheeze, shortness of breath, chest tightness, and cough	Cough, sputum production, wheezing, and chest tightness In severe cases: fatigue, weight loss, exercise intolerance, and anorexia
Pathophysiology	Airway narrowing caused by hyperresponsiveness, inflammation, structural remodeling, and environmental exposures	Airway narrowing caused by chronic inflammation as a response to cigarette smoke or noxious environmental stimuli, parenchymal tissue destruction, and small airway fibrosis; high prevalence of comorbidities

COPD: chronic obstructive pulmonary disease.

**Table 2:** Risk factors for poor asthma outcomes that increase the risk for exacerbation and may require stepped-up controller therapy.

Types of risk factors	Risk factors for exacerbations
Condition	(i) Uncontrolled asthma symptoms
Medications	(i) High SABA use (with increased mortality if >1 x 200-dose canister/month) (ii) Inadequate ICS or not prescribed ICS (iii) Poor adherence to ICS (iv) Incorrect inhaler technique
Comorbidities	(i) Obesity (ii) Chronic rhinosinusitis (iii) Gastroesophageal reflux disease (iv) Confirmed food allergy (v) Pregnancy
Exposures	(i) Smoking (ii) Allergen exposure if sensitized (iii) Air pollution (iv) Weather changes
Context	(i) Major psychologic or socioeconomic problems (ii) Low health literacy
Lung function	(i) Low FEV_1_, especially <60% predicted (ii) High bronchodilator reversibility
Other	(i) Other tests in patients with type 2 inflammation (e.g., blood eosinophils, elevated FeNO) (ii) Ever intubated or in ICU for asthma (iii) ≥1 severe exacerbations in last 12 months

FeNO: fractional exhaled nitric oxide; FEV_1_: forced expiratory volume in 1 second; ICS: inhaled corticosteroid; ICU: intensive care unit; SABA: short-acting beta agonist.
